# Pseudocholinesterase enzyme deficiency: a case series and review of the literature

**DOI:** 10.1186/1757-1626-2-9148

**Published:** 2009-12-04

**Authors:** Beyazit Zencirci

**Affiliations:** 1 Department of Anesthesiology and Reanimation, Mostas Private Health Hospital, Kahramanmaras, Turkey

## Abstract

**Introduction:**

Pseudocholinesterase (butyrylcholinesterase) is a drug metabolizing enzyme responsible for hydrolysis of the muscle relaxant drugs succinylcholine and mivacurium. Deficiency from any cause can lead to prolonged apnoea and paralysis following administration of succinylcholine and mivacurium.

**Case presentation:**

Within the last two years we have had four patients who have had prolonged apnea following the administration of mivacurium. It was understood that one was congenital and the other three due to various reasons had enzyme-deficiencies. In all four of the patients, the prolonged blocks deteriorated.

**Conclusion:**

Prolonged blocks may be encountered due to mivacurium use. The diagnosis of pseudocholinesterase enzyme deficiency can be given after a careful clinic supervision and peripheral nerve stimulator monitoring. A decrease in the activity of pseudocholinesterase enzyme and improvement in neuromuscular function will help verifying our diagnosis. Instead of pharmacological applications that may further complicate the situation, what should be done in such patients is to wait until the block-effect goes down by the help of sedation and mechanical ventilation.

## Introduction

Pseudocholinesterase (PChE) is an enzyme with a complex molecular structure [1]. It is synthesized in the liver and immediately released into the plasma [2]. The plasma half-life has been estimated to be approximately 12 days [3]. Deficiency or reduced activity of this enzyme results in significant prolongation of mivacurium or succinylcholine induced neuromuscular blockade [4]. In addition, PChE activity may be reduced by a number of disease states or by concomitant drug administration.

Mivacurium, which is a nondepolarizing neuromuscular blocking drug administered in doses of 0.1 to 0.2 mg/kg, also produces rapid onset of neuromuscular blockade lasting 15 to 30 minutes [5]. The rapid ester hydrolysis of mivacurium by PChE results in the short duration of action of this drug, which is ideal for providing muscle relaxation for brief surgical procedures [6]. But, the duration of mivacurium in adults is inversely related to serum PChE activity [7].

In this article, we would like to share our experiences in the management of four patients who developed mivacurium apnea postoperatively due to congenital or acquired pseudocholinesterase enzyme deficiencies alongwith a literature review.

## Case presentation*

### Patient 1

A 31-year-old Turkish woman, weighing 78 kg, was scheduled for caesarean section under general anesthesia. She was not operated previously. Induction of anesthesia was achieved with 130 mg of propofol. Muscular relaxation was achieved before intubation with 12 mg of mivacurium. Isoflurane was used as the general anesthetic inhalation agent. The operation lasted for 30 minutes and the sectio-surgery was uneventful. After the surgery, the inhalation agent was discontinued and the patient received 100% oxygen. It was noted that emergence seemed to be prolonged after 10 minutes. All vital signs were stable, showing no signs of tachycardia or hypertension. Oxygen saturation remained 100%. After an additional 10 minutes, there was suspicion of a PChE deficiency. Peripheral nerve stimulator (PNS) produced zero twitches. Three milligrams of midazolam was administered intravenously for its sedation and amnestic effects. Later the patient was transferred to the post-anesthesia Care Unit (PACU) for observation and ventilator support. Sixty-two minutes later, spontaneous muscle twitching was noted. One hour and twenty-two minutes later from the initial use of mivacurium, the patient had regained sufficient motor function to meet extubation requirements. Blood samples were drawn and sent to confirm a PChE deficiency [Paitent's PChE value (normal range) 1017 IU/L (2000 to 11000 IU/L)]. The patient was transferred to a hospital ward for the evening and discharged two days later. The PChE values of the patient who was called for a control after two months was considered between the normal ranges (3124 IU/L).

### Patient 2

A 47 year-old Turkish male, weighing 83 kg, was scheduled for laparoscopic cholecystectomy under general anesthesia. The patient had received two previous general anesthetics one for appendectomy 28 years ago and another for right inguinal hernia operation 19 years ago. It was learned that the case was applied succinylcholine during both of her previous operations and that he did not have a a history of a post-operative apnea etc. The patient had been using sertraline (100 mg/day) for 3 years due to major depressive disorder. Induction of anesthesia was achieved with 200 mg of propofol. Tracheal intubation was achieved with 17 mg of mivacurium. Isoflurane was used as the general anesthetic inhalation agent. The operation lasted for 25 minutes and further dose(s) of mivacurium were not required. After the surgery, the inhalation agent was discontinued, the patient received 100% oxygen but PNS produced zero twitches. All vital signs were stable, and oxygen saturation remained 100%. After an additional 20 minutes, there was suspicion of a PChE deficiency. Five milligrams of midazolam was administered intravenously for its sedative and amnestic effects. Later the patient was transferred to the PACU for observation and ventilator support. Two hours and twenty minutes later, spontaneous muscle twitching was noted. Three hours and fifteen minutes later from the initial use of mivacurium, the patient had regained sufficient motor function to meet extubation requirements. Plasma cholinesterase was found to have very low activity (788 IU/L). The patient was transferred to a hospital ward for the evening and discharged four days later.

### Patient 3

A 73 year-old Turkish woman, weighing 43 kg, was scheduled for breast biopsy under general anesthesia. She had a cholecystectomy 32 years ago, a hysterectomy 23 years ago and an appendectomy operation 17 years ago. It was learned from the anamnesis of the patient that succinylcholine had been administered on three previous occasions during anesthesia without a prolonged effect. Following preoxygenation, induction of anesthesia was achieved using propofol 90 mg and later mivacurium 7 mg. Following tracheal intubation anesthesia was maintained with isoflurane. The procedure was completed within 20 minutes. But there was no clinical evidence of recovery of neuromuscular function and there was no response to train of four stimulation. Anesthesia was therefore maintained with isoflurane 0.5% in 50% nitrous oxide and oxygen. Because there was no evidence of improved neuromuscular function 60 minutes after administration of mivacurium. The patient was transferred to the PACU for continued mechanical ventilation and neuromuscular monitoring. Sedation was maintained with a continuous iv infusion of midazolam. Four hours and twenty minutes after the administration of mivacurium, train of four response ratio was 75%. Midazolam infusion was discontinued and clinical assessment confirmed recovery of neuromuscular function. Patient was extubated 340 minutes following the administration of mivacurium and further recovery was uneventful. Plasma cholinesterase was found to have very low activity (598 IU/L).

### Patient 4

A 28 year-old Turkish male, weighing 76 kg, was scheduled for unilateral varicocelectomy and hydrocelectomy under general anesthesia. The patient did not have an operation previously. Induction of anesthesia was achieved with 200 mg of propofol. Tracheal intubation was achieved with 15 mg of mivacurium. Isoflurane was used as the general anesthetic inhalation agent. The operation was completed within 30 minutes. After the surgery, the inhalation agent was discontinued and the patient received 100% oxygen. All vital signs were stable, oxygen saturation remained 100%, but PNS produced zero twitches. After an additional 10 minutes, there was suspicion of a PChE deficiency. Three milligrams of midazolam was administered intravenously for its sedative and amnestic effects. Fifty-five minutes later, spontaneous muscle twitching was noted. One hour and five minutes after the initial use of mivacurium, the patient had regained sufficient motor function to meet extubation requirements. Plasma cholinesterase was found to have low activity (961 IU/L). The patient was transferred to a hospital ward for the evening and discharged two days later.

*(Monitoring included electrocardiography (ECG), noninvasive arterial blood pressure (NIBP), oxygen saturation (SpO_2_), capnography (ETCO_2_), and peripheral nerve stimulator monitoring applied over the ulnar nerve in all four patients)

## Discussion

Mivacurium is a potent benzylisoquinoline, nondepolarizing neuromuscular blocking drug. A recommended intravenous dose of 0.15 to 0.2 mg/kg provides tracheal intubating conditions within 2 to 2.5 minutes, with a predicted duration of action of 15 to 25 minutes, making it an ideal drug for short procedures requiring tracheal intubation [8]. İt is a structural relative of atracurium, but it does not undergo Hofmann elimination and is rapidly hydrolysed by PChE. Its duration of action is affected by the activity of this enzyme.

PChE is a tetrameric glycoprotein enzyme produced by the liver that hydrolyzes choline esters, such as those found in succinylcholine, mivacurium, procaine, chloroprocaine, tetracaine, cocaine and heroine [9]. In patients with a normal genotype for PChE, mivacurium's duration of action is inversely related to PChE activity and duration of action is slightly prolonged if activity is low [10]. Reduced PChE activity may occur as a result of inherited causes related to mutations at a single autosomal location on the long arm of chromosome 3 [11].

When there is a deficiency of this enzyme due to the presence of one or more atypical alleles, the mivacurium is not properly metabolized and thus, muscle paralysis can last for several hours. There are two forms of inherited atypical pseudocholinesterase deficiency. The heterozygous atypical form affects anywhere from 1 in 25, to 1 in 480 individuals (depending on the severity of the condition) [9]. Patients heterozygous for an abnormal enzyme may show up to 50% prolongation of block [12]. The homozygous atypical form affecting approximately 1 in 3200 to 5000 individuals [9]. In patients homozygous for an abnormal enzyme duration may be significantly prolonged, and even a small dose, (eg.0.03 mg/kg) can result in complete paralysis for up to 128 minutes [13]. Patient 4 did not have an operation previously as well, he also did not have a negative medical family history and he did not accept our spinal anaesthesia offer. Block time was prolonged by 30-35 minutes which did not require a post-operative care unit. Enzyme level was found to be slightly low (961 UI/L). And the case was considered as heterozygote atypic enzyme defected.

PChE activity may be affected by different disease states and/or by drug administrations. Physiologic reductions may occur with extremes of age and during pregnancy [14]. The low enzyme value (1017 IU/L) of Patient 1 which was measured during her cesarean section operation was found to be within the normal ranges in the laboratory assessment carried out during the control after two months (3124 IU/L) which proved us right in thinking that the PChE deficiency was due to pregnancy.

Other acquired causes of decreased activity include renal and liver disease, malignancy, burns, chronic debilitation/malnutrition, myocardial infarction/cardiac failure, collagen diseases, myxedema, and organophosphate poisoning [14]. Patient 3 previously had three operations under general anaesthesia by using succinylcholine. Renal function was normal in this patient. Liver function tests exposed a reduced albumin level (2.1 g/dL = 21 g/L), consistent with the diagnosis of malnutrition that was evident from patient's general cachectic appearance (weighing only 43 kg), and history of reduced caloric intake and recent weight loss. However, presence of a prolonged apnea and low enzyme activity (598 IU/L) in this operation although the case had not had any problems during the previous operations led us to think that this was a malnutrition-associated PChE deficiency. Actually, plasma cholinesterase levels in human subjects with protein energy malnutrition has been investigated [15]. PChE deficiency secondary to malnutrition has also been reported in patients with active Crohn's disease [16], and anorexia nervosa [17].

In addition, drugs which inhibit the enzyme's activity include acetylcholinesterase inhibitors (neostigmine, pyridostigmine, physostigmine, and edrophonium) anticholinesterases (especially echothlophate), cytotoxic agents (such as cyclophosphamide), steroids, ester-type local anaesthetics, hexafluorenium, pancuronium and oral contraceptives [18].

Sertraline is a serotonin reuptake inhibitor (SSRI) that is indicated for the treatment of depression. With therapeutic doses, side effects are minimal. Sertraline has a wide therapeutic index and appears to be safer than the tricyclic antidepressants in overdose [19]. On the other hand, the usual clinical antidepressants (fluoxetine, sertraline, and amitriptyline) are inhibitors of the cholinesterases on human serum and erythrocyte membrane. The order of inhibitory potency was sertraline> amitriptyline>>fluoxetine [20]. Our Patient 2 patient had been using sertraline (100 mg/day) for 3 years due to depression. He previously had three operations under general anaesthesia by using succinylcholine. However, presence of a prolonged apnea and low enzyme activity (788 IU/L) in this operation, although the case had not had any problems during the previous operations led us to think that this was a sertaline-associated acquired PChE deficiency. Besides, the re-tested (3 months after the operation) PChE value (2762 IU/L) was seen to have turned back to normal after the patient gave up using sertraline under psychiatrist control. We did not discover a case that implies an interaction between sertraline and pseudocholinesterase in our literature review. We believe this is the first case presentation in that sense.

Mivacurium should probably not be used for any patient who gives a history of prolonged block after succinylcholine. Any patient who experiences prolonged block after mivacurium should be advised on the need to have their own and their immediate family's plasma cholinesterase status checked. It was detected from the blood-samples received from the first-degree relatives of Patient 4 that PChE activities of them were also lower than 900 IU/L, and however that incidentally none of them had operation histories.

The clinician should be aware that if they have administered neostigmine to a patient with prolonged mivacurium-induced block, then the patient's plasma cholinesterase activity will be inhibited. Therefore, blood should not be drawn for cholinesterase activity until the effect of neostigmine has dissipated. As for four of our cases, by the time PChE defect suspicions alongwith PNS findings arose, we send blood samples to laboratory for enzyme values. We supported the cases for degradation of mivacurium with present enzyme activities solely with sedation, mechanical ventilation and PNS monitorization and without neostigmine application.

## Conclusion

We may find out that prolonged blocks can be encountered as a result of PChE enzyme defect when mivacurium is used. This situation can either be from congenital as in one of our patients (Patient 4) or due to various resons (pregnancy, malnutrition and sertraline use) as in the other three patients.

In these situations, attempted reversal of the block was at best only partially successful, and the patients were partially paralysed and minimally sedated while attempts were made to have them breathe; a very unpleasant experience. In the cases of prolonged block, no reversal was attempted, the cases were kept sedated and their lungs mechanically ventilated until the block had recovered spontaneously, and then extubated without problems. This patient had a much less traumatic experience than the others, in whom pharmacological reversal was attempted.

## Abbreviations

PchE: pseudocholinesterase; PNS: peripheral nerve stimulator; PACU: post-anesthesia care unit.

## Consent

Written informed consent was obtained from the patients for the publication of this case report and accompanying images. A copy of the written consent is available for review by the Editor-in-Chief of this journal.

## Competing interests

The author declares that they have no competing interests.

## Authors' contributions

BZ presented the case history, performed case management, drafted the manuscript. The author read and approved the final manuscript.
